# Open or laparoscopic treatment for hydatid disease of the liver? A 10-year single-institution experience

**DOI:** 10.1007/s00464-012-2719-0

**Published:** 2013-01-31

**Authors:** Florin Zaharie, Dana Bartos, Lucian Mocan, Roxana Zaharie, Cornel Iancu, Claudiu Tomus

**Affiliations:** 1 Department of Surgery, Iuliu Hatieganu, University of Medicine and Pharmacy, Octavian Fodor Regional Institute of Gastroenterology and Hepatology, 19-21 Croitorilor Street, 400162 Cluj-Napoca, Romania; 2 Department of Gastroenterology, Iuliu Hatieganu, University of Medicine and Pharmacy, Octavian Fodor Regional Institute of Gastroenterology and Hepatology, 19-21 Croitorilor Street, 400162 Cluj-Napoca, Romania

**Keywords:** Hepatic hydatid cyst, Laparoscopic surgery, Treatment of hydatid disease

## Abstract

**Background:**

Selection of the most appropriate treatment to obtain the lowest morbidity, mortality, and recurrence rates is mandatory for hydatid disease of the liver. This study evaluated the results of laparoscopic treatment (compared with the open approach) in the context of a 10-year single-institution experience.

**Methods:**

Between January 1998 and January 2008, 333 patients with hydatid disease of the liver underwent surgery in the authors’ department. Only the following aspects were considered as selection criteria for laparoscopic surgery: liver cyst not located in segment 1 or 7, with corticalization on the surface and no evidence of intrabiliary rupture. Of 62 patients who underwent laparoscopic treatment, 3 required conversion to open surgery. The remaining 59 patients (group 1) were analyzed. During the same period, 271 patients with hepatic hydatid disease underwent conventional surgery, but only 172 records were compatible with the criteria for the laparoscopic approach and the respective patients were retrospectively reviewed (group 2).

**Results:**

Conversion to open surgery occurred in three cases (4.84 %). The mean cyst diameter was 6.62 cm (range, 2–15 cm) in group 1 and 7.23 cm (range, 2–18 cm) in group 2 (*p* = 0.699). The mean operative time was 72 min (range, 45–140 min) in group 1 and 65 min (range, 35–120 min) in group 2 (*p* < 0.001). The general complication rate and abdominal wound complication rate were respectively 0 % and 0 % in group 1 (*p* = 0.023) compared with 5.23 and 8.72 % in group 2 (*p* = 0.015). The mean hospital stay was 6.42 days (range, 1–21 days) in group 1 and 11.7 days (range, 4–80 days) in group 2 (*p* < 0.001). The mean follow-up period was 24.2 months (range, 6–32 months) in group 1 and 28.4 months (range, 6–40 months) in group 2. No recurrences were observed in either group during this period.

**Conclusion:**

Laparoscopic surgery provides a safe and efficacious approach for almost all types of hepatic hydatid cysts. Large, prospective, randomized trials are needed to confirm its superiority.

Hydatid disease is a severe parasitic disease with a widely ranging distribution. Echinococcosis is considered to be endemic in regions wherein farming is the basic occupation of the population [1].

Although liver hydatidosis is considered a benign disease, it has a considerable social and economic impact. Without treatment, the cysts grow in size and eventually cause complications leading to disability or even exitus. Only in exceptional circumstances can spontaneous healing occur through the parasite’s death and calcification. For these reasons, it is generally accepted that hydatid disease must be treated once it is diagnosed. Surgery remains the gold standard therapy [2, 3] despite the increased interest in nonsurgical techniques. Because the open procedures are followed by significant morbidity, especially in terms of wound infection [2, 3], the laparoscopic approach has become increasingly popular, although controversies regarding the role of laparoscopy in the management of hydatid disease have not been resolved to date [2].

This study presents the results of both open and laparoscopic treatment in the context of a 10-year single-institution experience.

## Patients and methods

Between January 1998 and January 2008, 333 patients with hydatid disease of the liver underwent surgery in our department. Only the following aspects were considered as selection criteria for laparoscopic surgery: liver hydatid cysts not located in segment 1 or 7 of the liver (Couinaud’s segmentation), with corticalization on the surface of the liver and no evidence of intrabiliary rupture.

Intrabiliary rupture was suspected preoperatively in the following cases:

1. Liver hydatid cyst with Gharbi type 2 during abdominal ultrasound associated with hepatic cytolysis and cholestasis changes.
2. Liver hydatid cyst with Gharbi type 2 or 4 during abdominal ultrasound associated with the presence of jaundice during hospital admission or in the medical history.
3. Liver hydatid cyst with Gharbi type 2 or 4 during abdominal ultrasound, with common bile duct dilation exceeding 10 mm and elevated cholestatic levels.
4. Liver hydatid cyst with Gharbi type 2 or 4 during abdominal ultrasound and the presence of echogenic material within the common bile duct.

All the respective patients underwent preoperative endoscopic retrograde cholangiopancreatography (ERCP). Patients showing a communication between the cystic tumor and the bile duct were excluded from the laparoscopic group. Of 333 patients, 62 underwent laparoscopic treatment. Three of these patients required conversion to open surgery and were excluded from the study. The remaining 59 patients (group 1) were retrospectively analyzed. All the patients were treated with albendazole (400 mg twice a day or 12 mg/kg when the weight was <60 kg) before the operation (4–7 days).

### Surgical techniques for group 1

Four ports were placed as follows: a supraumbilical 10 mm port through which a 30° telescope was inserted, a 10 mm port inserted at the epigastrium as near as possible to the cyst and used as a working channel, and two 5 mm ports inserted according to cyst location. The abdominal cavity was insufflated with carbon dioxide, and any adhesion between the cysts and the neighboring organs was lysed.

Next, the hydatid lesions were isolated from the remainder of the peritoneal cavity through wicks soaked in 20 % hypertonic saline solution. The tip of a puncture cannula was pushed inside the cystic cavity, injecting 20 % hypertonic saline solution. Another vacuum cannula, inserted through the other working trocar, was permanently maintained in the vicinity of the puncture point to prevent any hydatid spillage.

After 5 min, the hydatid content was aspirated. Starting from the puncture site, cystotomy was practiced, and the germinal membrane together with the soaked wicks and the sectioned pericyst were extracted in a plastic bag (endobag). After parasite inactivation and removal, the surgical procedures chosen for the laparoscopic treatment of the residual cavity were Lagrot partial pericystectomy (and drainage of the remaining cavities) (54 cases) and total pericystectomy (5 cases).

Lagrot partial pericystectomy involves resection of the corticalized pericyst (externalized extrahepatic) up to the border with the liver parenchyma. After this procedure, the part of the intrahepatic pericyst (residual cavity) communicating with the remainder of the peritoneal cavity remains in situ. The five cases solved by total pericystectomy required total excision of the pericyst after prior inactivation of the hydatid content.

In two cases, the presence of a biliocystic fistula was detected (small fistulous orifice) during surgery. This situation was solved by applying a metal clip and performing a suture (X-wire at this level). When occult cystobiliary fistula was suspected (avital hydatid cysts or secondary infected cysts present during abdominal ultrasound as a heterogeneous mass) but biliocystic communication could not be visualized intraoperatively, the choice after Lagrot partial pericystectomy was double external drainage of the residual cavity. Later, if necessary, ERCP was performed to decrease the pressure in the biliary tract (see the Postoperative morbidity in group 1 section later).

During the same period, 274 patients with hepatic hydatid disease underwent conventional surgery (including the 3 patients who required conversion to open surgery). Of the 274 patients, 69 presented with intrabiliary rupture, eight presented with spontaneous rupture into the peritoneal cavity, and 25 had the cysts located in segment 7 of the liver. These patients were excluded from the study. The remaining records for 172 patients who met the criteria for the laparoscopic approach were retrospectively reviewed (group 2).

### Surgical techniques for group 2

For the open surgical approach, we used a supraumbilical midline incision or a subcostal incision. Any adhesion between the cysts and the neighboring organs was lysed. To prevent secondary peritoneal hydatidosis, the peritoneal cavity was isolated with wicks soaked in 20 % hypertonic saline solution before any maneuver on the hydatid cyst was performed. Parasite inactivation was performed by injecting 20 % hypertonic saline solution.

After 5 min, the hydatid content was aspirated. Starting from the puncture site, cystotomy was performed, with extraction of the germinal membrane and daughter vesicles. The surgical procedures chosen for open treatment of the residual cavity were Lagrot partial pericystectomy and drainage of the remaining cavities (136 cases), total pericystectomy (26 cases), left lobectomy (Couinaud’s classification; 9 cases), and left hepatectomy (Couinaud’s classification; 1 case).

### Statistical analysis

All statistical analyses were performed using the SPSS 17 software package. Statistical comparative analyses were performed using the *χ* ^2^ test and the *t* test. A *p* value lower than 0.05 was considered to denote statistical significance.

## Results

### Demographic data and concurrent comorbidities

Detailed demographic data, concurrent comorbidities, and the preoperative risk profile of both surgery study groups are presented in Table 1. Both groups were similar in terms of age, gender, overall concurrent comorbidities, and preoperative risk profile (American Society of Anesthesiologists [ASA] classification). Obese or overweight patients were more numerous in group 2 (treated by the classic approach) (24.42 vs. 10.17 %; *p* = 0.032), although more than 10 % of the patients treated by laparoscopic approach presented with these concurrent comorbidities.

**Table 1:** Demographic data and concurrent comorbidities in the patient population

Parameter	Laparoscopic group		Conventional group		*p* value
	(*n* = 59)	(%)	(*n* = 172)	(%)	
Age (years)					
<50	48	81.35	136	79.06	0.851
>50	11	18.65	36	20.94	
Mean age (years)	43.8 ± 8.3		45.7 ± 7.9		0.117
Sex					
Female	31	52.54	103	59.88	0.405
Male	28	47.46	69	40.12	
Concurrent comorbidities	0.362				
Diabetes mellitus	6	10.17	24	13.95	0.602
High blood pressure	14	23.73	54	31.40	0.342
Ischemic heart disease	13	22.03	35	20.35	0.929
Overweight or obesity	6	10.17	42	24.42	0.032^a^
ASA	0.092				
1–2	28	47.45	57	33.14	0.070
3	27	45.77	91	52.90	0.427
4	4	6.78	24	13.96	0.219

^a^ *p* < 0.05 (statistically significant difference)

### Intraoperative characteristics of the cysts and perioperative morbidity and mortality

The pathologic characteristics of the cysts and the surgical procedures used for the treatment of the hepatic hydatid cysts in both surgery study groups are presented in Table 2. The average size of the liver hydatid cysts was 6.62 cm (range, 2–15 cm) in group 1 and 7.23 cm (range, 2–18 cm) in group 2. Both groups were similar in terms of cyst location, size, and type (character), as well as the surgical procedures used for treatment (although Lagrot partial pericystectomy was used more frequently in group 1 than in group 2: 91.52 versus 78.5 %, *p* = 0.041).

**Table 2:** Pathologic features of the cysts and the surgical procedures used in the patient population

Parameter	Laparoscopic group		Conventional group		*p* value
	(*n* = 59)	(%)	(*n* = 172)	(%)	
Location of the cyst (Couinaud’s classification)	0.366				
Segments 2–4	14	23.72	51	29.65	0.480
Segments 5–6	24	40.68	53	30.82	0.220
Segment 8	21	35.60	68	39.53	0.707
Size of the cyst (maximum diameter) (cm)	0.699				
<5	13	22.03	30	17.44	0.556
5–10	41	69.50	124	72.10	0.830
>10	5	8.47	18	10.46	0.850
Type (character) of the cyst	0.550				
Pure clear fluid cyst (Gharbi type 1)	23	38.98	53	30.82	0.322
Hydatid daughter cyst (Gharbi type 3)	16	27.11	68	39.53	0.120
Calcified (Gharbi type 5)	7	11.87	19	11.05	0.947
Avital hydatid cyst (heterogeneous mass)	6	10.17	15	8.72	0.942
Secondarily infected cyst	7	11.87	17	9.88	0.853
Surgial procedures used	0.127				
Lagrot partial pericystectomy	54	91.52	136	78.5	0.041^a^
Total pericystectomy	5	8.48	26	15.11	0.285
Left lobectomy	0	0	9	5.21	0.162
Left hepatectomy	0	0	1	0.58	0.572

^a^ *p* < 0.05 (statistically significant difference)

**Table 3:**
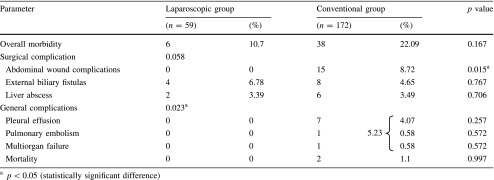
Postoperative morbidity and mortality

Conversion to open surgery occurred in three cases (4.84 %). The main reasons for conversion to open surgery were bleeding (2 cases) and difficult location of the cyst (inadequate exposure; 1 case).

The mean operative time was 72 min (range, 45–140 min) in group 1 and 65 min (range, 35–120 min) in group 2 (*p* < 0.001). The mortality rate was 0 % for group 1 and 1.16 % (2 cases) for group 2. The difference between the two groups was not significant (*p* = 0.997).

Although the overall morbidity rate was 10.7 % (6 cases) in group 1 and 22.09 % (38 cases) in group 2, with no significant difference between the two groups (*p* = 0.167), the statistical analyses of the postoperative outcome showed that the incidence of wound complications (seroma and abscess) and general complications (pleural effusions, pulmonary embolism, organ failure) were significantly higher for the open group (group 2: 8.72 and 5.23 %, *p* = 0.015) than for the laparoscopic group (0 and 0 %, *p* = 0.023) Table 3.

### Postoperative morbidity in group 1 (laparoscopic approach)

Group 1 had two abscesses of the residual cavity and four cases of external biliary fistulas. The hydatid cysts developing the two abscesses of the remaining cavity were medium-sized and located in segments 8 and 3. Both abscesses were laparoscopic ally drained.

Of the four hydatid cysts with postoperative development of biliary fistulas, only one was intraoperatively diagnosed as abscessed. The remaining three hepatic cysts were acephalocyst (with pure clear cyst fluid) or cysts with daughter vesicles. No biliocystic communication was detected intraoperatively in any of the cases. The flow of the four biliary fistulas significantly decreased after intestinal transit resumption, but they closed spontaneously in only two cases. The remaining two cases needed ERCP with endoscopic sphincterotomy, which accomplished closure of the biliary fistulas in 7–10 days.

### Postoperative morbidity in group 2 (conventional approach)

Most of the surgical complications in group 2 were wound complications (seromas, suppuration: 8.72 %, 15 cases) and biliary fistulas (4.65 %, 8 cases). Wound complications (seromas or suppuration of the wound) required removal of two or three cutaneous stitches and collection evacuation followed by daily antiseptic treatment, with a favorable evolution.

Most of the patients (6 cases) who experienced postoperative biliary fistula were treated conservatively. The amount of bile drained through the drain tubes from the remaining cavity decreased dramatically after bowel transit resumption, with complete closure of the biliary fistula in 4–8 days. For the two cases in which the biliary fistula did not close spontaneously, ERCP was performed together with sphincterotomy, with closure of the biliocystic fistula accomplished in 5 days, from labor in one case. The remaining case had a slow unfavorable evolution with septic hepatic abscess, which required laparotomy.

In six cases (3.49 %), hepatic abscess developed in the remaining cavity. Two of the cases required surgical intervention with abscess evacuation, cavity cleansing, and multiple drainage, which resulted in a favorable evolution. For the third case, right hepatectomy was performed, but the patient experienced septic shock and multi-organ failure and died on the 60th day of hospitalization.

Six of the seven cases with pleural collections did not require puncture with evacuation but were treated conservatively with antibiotics and nonsteroidal antiinflammatory drugs (NSAIDs). A single case required pleural puncture, with 350 ml of serocitrin liquid extracted and the patient experiencing a favorable outcome.

### Hospital stay and evidence of hydatid recurrence

The mean hospital stay was 6.42 days (range, 1–21 days) in the laparoscopic group (group 1) and 11.7 days (range, 4–80 days) in the open group (group 2). The stay was significantly longer for group 2 (*p* < 0.001).

The mean follow-up period was 24.2 months (range, 6–32 months) for group 1 and 28.4 months (range, 6–40 months) for group 2. No recurrences were observed in either group during this period.

## Discussion

Although the possibilities for the treatment of hepatic echinococcosis have increased considerably in recent years (including medical treatment, PAIR, or a combination of these two), surgery remains the mainstay for healing of hydatid disease [2, 3]. Due to the development in technology and especially the increasing number of more experienced surgeons, laparoscopic surgery has been introduced for the surgical treatment of liver hydatid disease liver as well as for the surgical treatment of many other organs.

Initially, however, laparoscopy was not quickly accepted or widely used in the treatment of hydatid disease due to the concern that the recurrence rate and the risk of intraperitoneal dissemination might be higher with laparoscopy than with the conventional approach [4, 5]. Different authors have attempted to reduce the risks with laparoscopy by pre- and postoperative albendazole therapy, proper isolation of the cyst from the remainder of the peritoneal cavity (using various devices), and the use of wide-angle laparoscopes [6–8]. In fact, the real risk of spillage is lower than might be expected [9], and the short-term recurrence rate varies between 0 and 9 % after laparoscopy, whereas in open cases, it is higher (0–30 %) [10, 11].

Laparoscopic treatment of liver hydatidosis should not be regarded as a new surgical technique but rather as a new and minimally invasive access (with all its benefits) for performing a popularly established surgical intervention. Like any other surgical intervention, laparoscopic treatment of liver hydatidosis complies with the basic surgical principles of treating liver hydatid cysts by an open approach including prevention of hydatid spillage, sterilization and evacuation of the parasite, and management of the residual cavity [2–5].

Most of the reports on laparoscopic treatment of liver hydatidosis consist of case reports or small patient series [2, 5, 7, 12]. They could give the misleading impression that they are oriented to publish successful results with this technique, but the difference detected in favor of the minimally invasive approach could be due to the limited number of patients and the rigorous selection criteria (central location of the cyst, cyst size exceeding 10 cm, cysts with thickened and calcified walls).

Our series of 59 patients is one of the largest series in the literature, and our selection criteria were truly permissive (including any patient wanting a laparoscopic approach whose cyst was not communicating with the biliary tree or was located in liver segment 1 or 7). Our series included a large variety of hydatid cysts. Most of them were proligere cysts with daughter vesicles (>66 %), but infected or calcified cysts were represented as well. Regarding cyst size, although most cysts were medium-sized (5–10 cm), a large number of giant cysts (>10 cm) were treated by means of the laparoscopic approach.

Another great advantage of laparoscopic treatment is that the laparoscope can be inserted inside the cystic cavity, allowing its inspection. The image of the pericystic cavity’s interior displayed on monitors actually is two to three times larger. If a biliocystic communication is observed, it can be approached by applying a clip or an X-shaped wire. Also, remnants of the germinal membrane can be identified and removed, reducing the incidence of recurrence or suppurative complications.

A few disadvantages of the laparoscopic approach need to be considered. For example, laparoscopy still is limited in terms of liver resection, closure of biliary communications, and achievement of pericystodigestive anastomoses, although in recent years, an increasing number of authors have published promising results (small series of patients) [12–14].

We did not perform any hepatic resections or pericystodigestive anastomoses via laparoscopy, although a recently published review involving a large number of patients (1,294 patients with liver resection, 314 of whom were treated via laparoscopy) proved that laparoscopic liver resection is safe and feasible with definite short-term benefits and lower postoperative morbidity [15].

No prospective, randomized clinical trials comparing laparoscopic with open surgical treatment of hydatid disease have been reported. Postoperative morbidity ranges from 8 to 25 % in laparoscopic studies and from 12 to 63 % in open series [4]. Treatment-related death after laparoscopy is almost zero in laparoscopic series, whereas it ranges from 0 to 3 % in open series [4, 11].

Our morbidity rate was significantly lower in the laparoscopic group, mainly due to a lower incidence of abdominal wound complications (0 vs. 8.72 %, *p* = 0.015) and general complications (0 vs. 5.23 %, *p* = 0.023). No disease- or procedure-related mortality occurred in the minimally invasive treatment group. Similar results have been reported by other authors [8, 12].

Although the mean operative time was slightly longer with the laparoscopic approach (without statistical significance), we believe that this obstacle can easily be overcome by increased experience of the surgical team.

The encouraging results from the current study favor extending the limits of laparoscopy in hydatid disease, motivated primarily by a lower postoperative morbidity, an increased speed of healing, a shorter hospital stay, and superior aesthetic results. Knowing the relationship between the cyst and the biliary tree is essential in choosing the appropriate patients for the laparoscopic technique, although considering that laparoscopic hepatic resection is a growing option in the field of hepatic surgery [15], the only absolute contraindication to the laparoscopic approach in the treatment of liver hydatid cyst is posterior location of the cyst (segments 7 and 1). For surgeons experienced in liver surgery, working in centers with adequate technical equipment, the presence of biliocystic communication is a relative contraindication that can be overcome with increasing experience.

The indications for the laparoscopic approach in the treatment of liver hydatidosis have been and still are in constant change. It should not be forgotten that 15 years ago, the indications for a laparoscopic approach to the treatment of liver cyst were limited to small liver hydatid cysts (<5 cm) without daughter vesicles and in a peripheral location. All these contraindications proved to be overstated given that the same prophylactic measures are taken to reduce the risk of peritoneal hydatidosis and that the surgical time for the conventional surgery is observed. Therefore, the only real contraindication with absolute character is the surgeon’s inability to physically perform the suggested surgery (and this happens when the hydatid liver cyst has a posterior location: segments 7 and 1).

When the advantages of the laparoscopic approach are weighed, especially the fast healing and aesthetic results, which actually were the only real criteria for assessing the quality of the interventions, the disadvantages of minimally invasive approach are set aside. They are temporary impediments in perfecting the therapeutic concept of the minimally invasive approach, which surely will be the future of surgery.

## Conclusions

Many of the open surgery techniques for hepatic hydatid cysts can be performed laparoscopically, complying with the conventional tempo of the surgical intervention. Laparoscopic surgery provides a safe and efficacious approach to almost all types of liver hydatid cysts, but knowledge of the relationship between the cyst and the biliary tract is essential in choosing the appropriate patients. Considering the well-known benefits of minimally invasive surgery, the laparoscopic approach offers a viable alternative to conventional surgery for the treatment of liver hydatid cysts and is worthy to be considered for suitable situations.
